# Supplementary material for: Causal Relationship between Aging and Anorexia Nervosa: A White-Matter-Microstructure-Mediated Mendelian Randomization Analysis
Source: Biomedicines. 2024 Aug 16;12(8):1874. doi: 10.3390/biomedicines12081874 (PMC11351342; doi:10.3390/biomedicines12081874)
Supplement: Supplementary file 1 [file biomedicines-12-01874-s001.zip › Supplementary Figure/Figure S8/Leaveoneout plot of MR analysis for TL to ubm-b-1723.pdf]

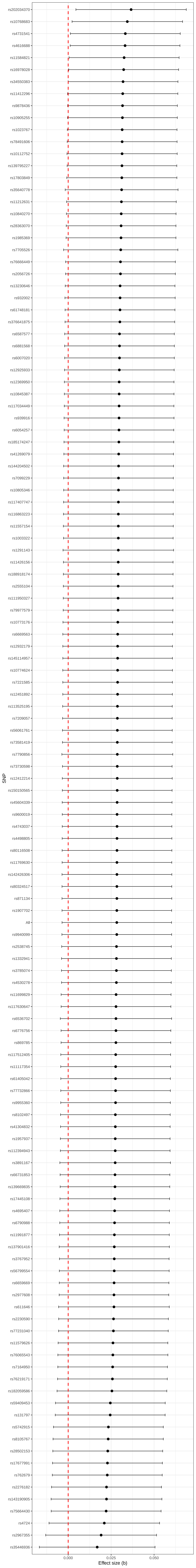

rs202034370
rs10768683
rs4731541
rs4616688
rs11584821
rs16978028
rs34550383
rs11412296
rs9878436
rs10905255
rs1023767
rs78491606
rs10112752
rs139795227
rs17803849
rs35640778
rs11212631
rs10840270
rs28363070
rs1985369
rs7705526
rs76666449
rs2056726
rs13230646
rs932002
rs61748181
rs376641875
rs6587577
rs6881568
rs6007020
rs12925933
rs12369950
rs10845387
rs117034449
rs939916
rs6054257
rs185174247
rs41269079
rs144204502
rs7099229
rs10805346
rs117407747
rs116863223
rs11557154
rs1003322
rs1291143
rs11426156
rs188918174
rs2555104
rs111950327
rs79977579
rs10773176
rs6669563
rs12932179
rs145114957
rs10774624
rs7221585
rs12451892
rs113525195
rs7209057
rs56061761
rs73581419
rs7790856
rs73730598
rs12412214
rs150150565
rs45604339
rs9600019
rs4743037
rs4498805
rs80116508
rs11769630
rs142426306
rs80324517
rs871134
rs1907702
All
rs9940099
rs2538745
rs1332941
rs3785074
rs4530278
rs11699829
rs117630647
rs6536702
rs6776756
rs869785
rs117512405
rs11117354
rs61405042
rs77732866
rs9955360
rs8102497
rs41304832
rs1957937
rs112394943
rs3891167
rs66731853
rs139669835
rs17445108
rs4695407
rs6790988
rs11991877
rs137901416
rs3767952
rs56799554
rs6659669
rs2977608
rs611646
rs2230590
rs77231040
rs11579626
rs76065543
rs7164950
rs76219171
rs182059586
rs59409453
rs131797
rs5742915
rs8105767
rs28502153
rs17677991
rs762679
rs2276182
rs143190905
rs75664430
rs4724
rs2967355
rs35446936
SNP
0.000
0.025
0.050
Effect size (b)
